# A randomised, non-inferiority study of chloroprocaine 2% and ropivacaine 0.75% in ultrasound-guided axillary block

**DOI:** 10.1038/s41598-021-89483-y

**Published:** 2021-05-11

**Authors:** Irene Sulyok, Claudio Camponovo, Oliver Zotti, Werner Haslik, Markus Köstenberger, Rudolf Likar, Chiara Leuratti, Elisabetta Donati, Oliver Kimberger

**Affiliations:** 1 Department of Anaesthesia, General Intensive Care Medicine and Pain Therapy, Medical University of Vienna, Vienna, Austria; 2 Department of Anesthesiology, Clinica Ars Medica, Genolier Swiss Medical Network, Gravesano, Switzerland; 3 Department of Gynecology, Medical University of Vienna, Vienna, Austria; 4 Department of Anesthesiology and Intensive Care Medicine, Klinikum Klagenfurt am Wörthersee, Klagenfurt, Austria; 5 CROSS Research SA, Mendrisio, Switzerland; 6 Sintentica SA, Mendrisio, Switzerland; 7 Outcomes Research Consortium, Cleveland, OH USA

**Keywords:** Drug development, Therapeutics

## Abstract

Chloroprocaine is a short-acting local anaesthetic with a rapid onset of action and an anaesthesia duration up to 60 min. In this pivotal study success rates, onset and remission of motor and sensory block and safety of chloroprocaine 2% was compared to ropivacaine 0.75% for short-duration distal upper limb surgery with successful block rates as primary outcome. The study was designed as a prospective, randomised, multi-centre, active-controlled, double-blind, parallel-group, non-inferiority study, performed in 4 European hospitals with 211 patients scheduled for short duration distal upper limb surgery under axillary plexus block anaesthesia. Patients received either ultrasound guided axillary block with 20 ml chloroprocaine 2%, or with 20 ml ropivacaine 0.75%. Successful block was defined as block without any supplementation in the first 45 min calculated from the time of readiness for surgery. 90.8% patients achieved a successful block with chloroprocaine 2% and 92.9% patients with Ropivacaine 0.75%, thus non-inferiority was demonstrated (10% non inferiority margin; 95% CI − 0.097, 0.039; p = 0.02). Time to onset of block was not significantly different between the groups. Median time to motor and sensory block regression was significantly shorter as was time to home discharge (164 [155–170] min for chloroprocaine versus 380 [209–450] for the ropivacaine group, p < 0.001). For short-duration surgical procedures, the short-acting Chloroprocaine 2% may be used, with success rates non-inferior to ropivacaine and a favourable safety profile.

**Trial registration: **The trial was registered at Clinicaltrials.gov with registration number NCT02385097 (March 11th, 2015) and European Clinical Trial Database with the EudraCT number 2014-002519-40 (July 7th, 2015, Austria—BASG).

## Introduction

Local anaesthesia is employed successfully in increasing numbers in a wide variety of surgical procedures to produce regional blockades without impacting the consciousness of patients, and is in particular suited for ambulatory surgery^1^. With the practice of outpatient surgery steadily growing, the ideal local anaesthetic should provide a rapid onset plus adequate potency and duration of action, combined with a favourable safety profile and low risk of systemic toxicity. Furthermore, patients should be able to recover motor and sensory functions shortly after surgery, be dischargeable on the same day while maintaining manageable post-surgery pain and discomfort^2,3^.

In general, the selection of the local anaesthetic primarily depends on the duration of the planned procedure. Chloroprocaine has very short-acting properties and belongs to the amino-ester class of local anaesthetics, and is characterized by a rapid onset of action between 6 to 12 min and an anaesthesia duration up to 60 min^4^, depending on the amount used, possible use of supplements, and its route of administration. Ultrasound guided axillary block for brachial plexus anaesthesia is a very popular anaesthetic technique for hand and forearm surgery, with a low incidence of complications and high rate of success^5,6^. For this technique to be successful, deposition of local anaesthetic adjacent to four nerves (the median, radial, ulnar and musculocutaneous nerves) is required. Ultrasound visualization of target nerves, needle and injectate spread vs. nerve stimulation have been associated with improved block success rates, decreased block onset times and a decrease in the local anaesthetic dose needed^7,8^.

Despite the long history of chloroprocaine, detailed evidence on ultrasound-guided axillary plexus anaesthesia with this anaesthetic is lacking. Recently a new formulation of preservative-free Chloroprocaine HCl 2% (Sintetica SA, Switzerland) has been developed and has presently been licensed for use in regional anaesthesia in European countries. The present, pivotal study was designed to evaluate the non-inferiority regarding successful block rate and the safety of this formulation (Test product) compared to Ropivacaine HCl 0.75% (Naropin^®^, AstraZeneca GmbH, Germany; Reference product). Ropivacaine HCl 0.75% was chosen as the active control as it is the most commonly used regional anaesthetic in European countries in brachial plexus block procedures.

## Methods

This prospective, randomised, multi-centre, active-controlled, double-blind, parallel-group, non-inferiority pivotal study was approved by the local independent ethics commissions of each clinical centre (IRB Medical University of Vienna, Spitalgasse 23, 1090 Vienna, Austria; IRB Clinica Ars Medica, Via Cantonale, CH-6929 Gravesano, Switzerland; IRB Ospedale Regionale di Bellinzona e Valli-Bellinzona, CH-6500 Bellinzona, Switzerland, and IRB Kabeg Klinikum Klagenfurt am Wörthersee, Feschnigstrasse 11, 9020 Klagenfurt, Austria). It was classified as a phase III clinical trial, was registered at clinicaltrials.gov with registration number NCT02385097 (March 11th, 2015) and at the European Clinical Trial Database with the EudraCT number 2014-002519-40 (July 7th, 2015, Austria—BASG), conducted in accordance with International Council for Harmonisation Guidelines for Good Clinical Practice Good Clinical Practice and the declaration of Helsinki^9^. According to the protocol, 211 patients were enrolled between 01 April 2015 and 24 May, whose written, informed consent was obtained before enrolment. Since this was designed as pivotal study, the following guidelines of the European Medicine Agency were adhered to: “ICH E9 guideline on Statistical principles for clinical trials”, (http://www.ema.europa.eu/en/ich-e9-statistical-principles-clinical-trials) the “Guideline on the choice of the non-inferiority margin” (http://www.ema.europa.eu/en/choice-non-inferiority-margin) and the “Points to consider on switching between superiority and non-inferiority guideline” (http://www.ema.europa.eu/en/switching-between-superiority-non-inferiority).

The study was performed in the following 4 European clinical centres:

- Centre N. 1: Department of General Anaesthesia and Intensive Care Medicine, Medical University of Vienna, Spitalgasse 23, 1090 Vienna, Austria.
- Centre N. 2: Department of Anaesthesiology, Clinica Ars Medica, Via Cantonale, CH-6929 Gravesano, Switzerland.
- Centre N. 3: Department of Anaesthesiology, Ospedale Regionale di Bellinzona e Valli-Bellinzona, CH-6500 Bellinzona, Switzerland.
- Centre N. 4: Department of Anaesthesia, Intensiv-, Palliativ-, Pain Medicine, Kabeg Klinikum Klagenfurt am Wörthersee, Feschnigstrasse 11, 9020 Klagenfurt, Austria.

The *primary objective* of the study was to evaluate the non-inferiority of Chloroprocaine HCl 2%, Sintetica SA (Test) versus Ropivacaine HCl 0.75% (Naropin^®^, AstraZeneca; Reference) in terms of proportion of subjects with a successful block for distal upper limb surgeries. Successful block was defined as “anaesthesia adequate for the surgery” (defined as complete sensory block), without any supplementation in the first 45 min (even if surgery lasted for > 45 min), calculated from the time of readiness for surgery. Supplementation was defined as any i.v. pain medication or general anaesthesia or pre- or intra-operative systemic analgesia or additional local anaesthetic infiltration.

The *secondary study objectives* were:

- To compare test and reference products in terms of the time to onset of sensory block (readiness for surgery), time to regression of sensory block, time to onset and regression of motor block, need for supplemental anaesthesia/analgesia and time to eligibility for home discharge;
- To evaluate the safety and tolerability profile of the study treatments.

Only patients scheduled for short duration (< 60 min) distal upper limb surgery possible under axillary plexus block anaesthesia, with a BMI of ≥ 18 ≤ 32 kg m^−2^ and ≤ 32 kg m^−2^ and ASA I to III, were enrolled.

The patients were randomised to one of 2 treatment groups to receive either 20 ml Chloroprocaine HCl 2% or 20 ml Ropivacaine HCl 0.75% as anaesthetic before surgery. Allocation was performed in a 1:1 ratio according to a computer generated randomisation list. The randomisation list was computer-generated using the PLAN procedure of the SAS^®^ system version 9.3 (SAS Institute Inc., Cary, NC, USA).

### Blinding

All clinical staff members involved in perioperative procedures as well as the patients themselves were blind with respect to the administered treatment with the study medication being prepared by an unblinded staff member otherwise not involved in the study.

### Ultrasound guided anaesthesia procedure

Axillary block was performed under ultrasound guidance: the median, ulnar, radial and musculocutaneous nerves were identified, then a 5 cm needle was inserted towards the 4 nerves. Once appropriate perineural needle placement was visualized, the volume (20 ml) of investigational anaesthetic was incrementally injected perineurally and equally between the nerves without preference for surgical field.

### Measurements

Evaluations were performed individually for each nerve for sensory and motor block initially every 5 min after administration of regional anaesthesia until the patient was ready for surgery. Again, blocked limbs were evaluated as soon as possible *after* surgery, then every 15 min for the first hour, every 30 min for the next 2 h and finally hourly until regression.

*Sensory block assessments* (thermal perception and sensitive perception) were dichotomized as being present (score 1) or absent (score 0). Readiness for surgery was defined as an absent cold *and* touch sensation in *all* 4 nerve territories (complete sensory block).

*Motor block* was evaluated through specific motor tests for each nerve territory and scored according to the modified Bromage scale^10^. Onset of motor block was defined when a motor block score ≤ 2 on the modified Bromage scale was present in ≥ 3 nerve territories. However no motor block was required for successful block and/or readiness for surgery.

*Regression* of sensory block was defined to have occurred when cold sensation and sensitive perception had returned in at least one nerve territory. Regression of motor block was deemed to have occurred when motor score was ≥ 3 in at least one nerve territory.

*Discharge criteria* were defined as a score ≥ 18 on the modified Aldrete’s scoring scale^11^ and no feeling of pain.

*Safety* of the investigational products was assessed by evaluating treatment-emergent adverse events, neurological symptoms, ECGs and vital signs (blood pressure and heart rate). A neurologic symptoms questionnaire was performed on day 1 postoperatively and via phone on day 7. Adverse events were monitored from the screening visit, immediately after informed consent signature, up to the telephonic follow-up. Particular attention was given to systemic and local toxicity symptoms, neurological symptoms (paraesthesia, motor function problems and pain at the injection site) and allergic reactions. The study schedule is attached as electronic supplement 1.

### Statistics

#### Sample size considerations

Considering a one-sided type I error α = 0.025, a type II error β = 0.15, a non-inferiority margin δ = − 0.1 and a proportion of success of 0.95 in both treatment groups, 86 patients per treatment group were calculated^12^.

The proportion of success of 95% in both treatment groups (i.e. πT = π R = 0.95) was set in accordance with previous evidence on success rates of ultrasound-guided axillary blocks^13–15^. The non-inferiority margin of 10% (i.e. δ = − 0.1) was set in accordance with the expected proportion of success and taking into consideration that the success rate observed before the advent of the ultrasound guided approach and clinically accepted at that time was of 85. Under the assumption of an exclusion rate from the Per Protocol Set of 15%, a sample size of at least 102 patients per group was calculated. Final sample size for the study was 211 patients in all 4 clinical centres.

### Analysis

The proportion of subjects with a successful block was compared between treatment groups in a binomial regression model, with both factors “group” and “centre” as fixed effects in the per protocol set. Non-inferiority of the Test in comparison with the Reference treatment was demonstrated if the lower limit of the 95% two-sided confidence interval (CI) of the difference between treatments was greater than the non-inferiority margin δ = − 0.1. Reasons for excluding subjects from the per protocol set were fully reviewed and documented during the blind review meeting before breaking study blinding.

The analysis was performed using the standard setting of SAS^®^ PROC GENMOD.

Times to onset of sensory block and motor block, times to regression of sensory and motor block, time to administration of rescue anaesthesia or rescue analgesia, and first post-operative analgesia and time to eligibility for home discharge were analysed using Kaplan–Meier curves and compared between treatment groups by log-rank test. Safety variables were analysed descriptively.

SAS^®^ system version 9.3 (SAS Institute Inc., Cary, NC, USA) was used for all calculations.

A p-value < 0.05 was considered statistically significant.

### Previous presentation

Preliminary data presented as poster presentation at the ASA 2019 (19.10.2019–23.10.2019, Orlando, FL, USA).

## Results

**Figure 1:**
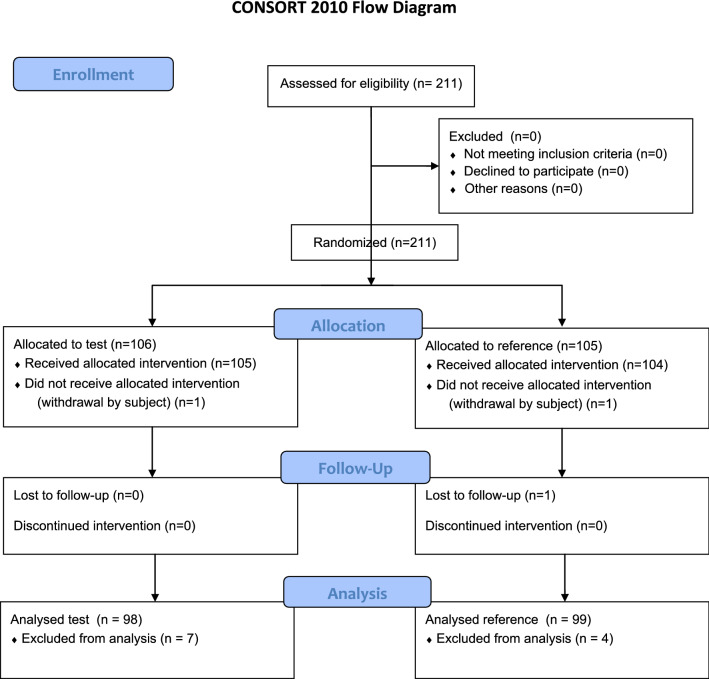
CONSORT flow diagram.

211 patients were enrolled in the study. 106 of the enrolled patients were randomised to the test treatment group and 105 patients to the reference treatment group. Two subjects, one in the test and one in the reference treatment group discontinued the study *before* treatment. In the chloroprocaine treatment group, 105 patients were treated and completed the study. In the ropivacaine treatment group, 104 patients were treated, of whom 103 completed the study with 1 patient lost to follow up. Primary efficacy analysis was conducted on the patients who completed the study according to the protocol without major deviations (see CONSORT Fig. 1). Demographic data is displayed in Table 1, surgery data can be found in Table 2.

**Table 1:** Demographic and morphometric data.

Demographic data	Chloroprocaine 2% N = 98	Ropivacaine 0.75% N = 99
**Sex**		
Female, n (%)	67 (68.4)	56 (56.6)
Male, n (%)	31 (31.6)	43 (43.4)
**Age (years)**		
Mean ± SD	55.7 ± 15.4	56 (56.6)
Median (range)	55.0 (21–89)	43 (43.4)
**Body weight (kg)**		
Mean ± SD	71.77 ± 14.02	72.97 ± 13.13
Median (range)	70.90 (45.0–125)	74.0 (46.0–106.0)
**Height (cm)**		
Mean ± SD	167.3 ± 8.6	169.0 ± 10.0
Median (range)	167.5 (145–198)	168.0 (150–192)
**BMI (kg/m**^**2**^**)**		
Mean ± SD	25.52 ± 3.75	25.45 ± 3.49
Median (range)	25.55 (18.9–31.9)	25.30 (18.0–32.0)
**Race**		
White, n (%)	97 (99.0)	95 (96.0)
Asian, n (%)	0 (0.0)	3 (3.0)
Others: Mestizo, n (%)	1 (1.0)	1 (1.0)

**Table 2:** Surgery data.

Surgical procedure	Safety set		
	Chloroprocaine 2% N = 105 N (%)	Ropivacaine 0.75% N = 104 N (%)	Overall N = 209 N (%)
Peripheral nerve decompression	24 (22.9)	25 (24.0)	49 (23.4)
Carpal tunnel decompression	20 (19.0)	6 (5.8)	26 (12.4)
Osteosynthesis	2 (1.9)	19 (18.3)	21 (10.0)
Ligament operation	7 (6.7)	12 (11.5)	19 (9.1)
Tendon sheath incision/- lesion excision	22 (21.0)	8 (7.6)	30 (14.4)
Synovectomy	6 (5.7)	5 (4.8)	11 (5.3)
Fasciectomy	6 (5.7)	3 (2.9)	9 (4.3)
Neurolysis	6 (5.7)	3 (2.9)	9 (4.3)
Tumour excision	1 (1.0)	6 (5.8)	7 (3.3)
Finger repair operation	3 (2.9)	3 (2.9)	6 (2.9)
Removal of foreign body	4 (3.8)	2 (1.9)	6 (2.9)
Bone operation	2 (1.9)	3 (2.9)	5 (2.4)
Hand repair operation	2 (1.9)	3 (2.9)	5 (2.4)
Cyst removal	3 (2.9)	1 (1.0)	4 (1.9)
Fracture treatment	3 (2.9)	1 (1.0)	4 (1.9)
Removal of internal fixation	1 (1.0)	3 (2.9)	4 (1.9)
Arthrodesis	0 (0.0)	3 (2.9)	3 (1.4)
Bone graft removal	3 (2.9)	1 (1.0)	3 (1.4)
Lipoma excision	2 (1.9)	1 (1.0)	3 (1.4)
Synovial cyst removal	1 (1.0)	2 (1.9)	3 (1.4)
Tendon operation/tenolysis	2 (1.9)	4 (3.9)	6 (2.8)
Trapeziectomy	0 (0.0)	3 (2.9)	3 (1.4)
Osteotomy	0 (0.0)	2 (1.9)	2 (1.0)
Peripheral nerve operation	1 (1.0)	1 (1.0)	2 (1.0)
Rheumatoid nodule removal	1 (1.0)	1 (1.0)	2 (1.0)
Autonomic ganglionectomy	1 (1.0)	0 (0.0)	1 (0.5)
Bone debridement	1 (1.0)	0 (0.0)	1 (0.5)
Bone lesion excision	0 (0.0)	1 (1.0)	1 (0.5)
Fascial operation	0 (0.0)	1 (1.0)	1 (0.5)
Finger amputation	0 (0.0)	1 (1.0)	1 (0.5)
Joint injection	1 (1.0)	0 (0.0)	1 (0.5)
Nail operation	1 (1.0)	0 (0.0)	1 (0.5)
Neurectomy	1 (1.0)	0 (0.0)	1 (0.5)
Scar excision	0 (0.0)	1 (1.0)	1 (0.5)
Skin implant	0 (0.0)	1 (1.0)	1 (0.5)
Varicose vein operation	0 (0.0)	1 (1.0)	1 (0.5)
Arthroscopy	0 (0.0)	2 (1.9)	2 (2.0)
Duration (mean (SD), min)	21.4 (13.5)	27.1 (13.5)	24.2 (13.9)

The proportion of subjects who achieved a successful block was 90.8% in the chloroprocaine group vs. 92.9% in the ropivacaine group. Non-inferiority of Chloroprocaine HCl 2% with respect to Ropivacaine HCl 0.75 was confirmed (p = 0.021; Table 3). No significant differences between treatments, or for hospitals or hospital-treatment-interactions were present.

**Table 3:** Block success.

Block success—non-inferiority test						
Patients proportion n (%)		LS means estimates		Difference	95% CI	One-sides p-value
Chloroprocaine 2%	Ropivacaine 0.75%	Chloroprocaine 2%	Ropivacaine 0.75%			
89 (90.8%)	92 (92.9%)	0.885	0.914	− 0.029	− 0.097, 0.039	0.0210

The lower limit of the 95% two-sided confidence interval of the difference between the two treatments proportion of success was above the pre-established 10% non-inferiority margin (δ = − 0.1) with clinical significance.

**Figure 2:**
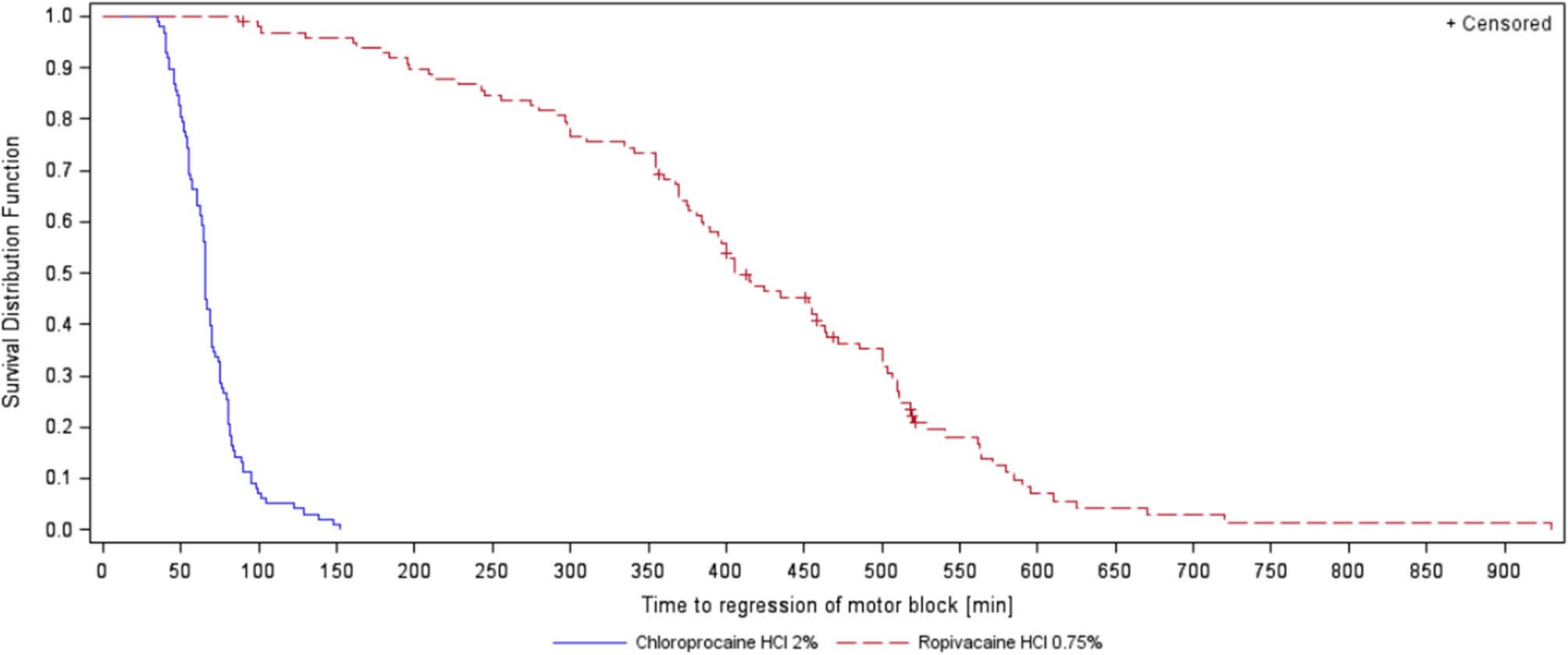
Time to regression of motor block (min).

**Figure 3:**
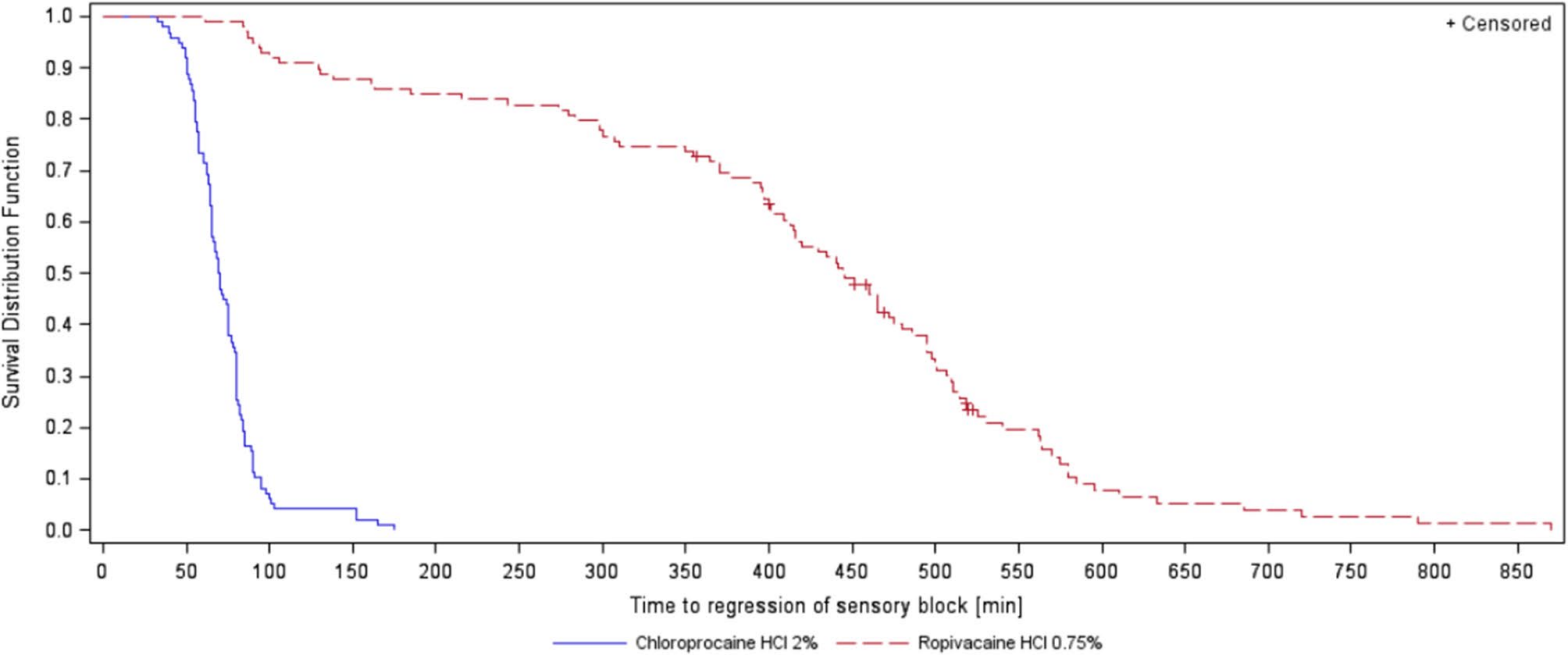
Time to regression of sensory block (min).

Analysis of onset and regression of sensory and motor block are displayed in Table 4. No significant differences were found for onset of sensory or motor block between the groups, although there was a distinct tendency for a more rapid onset of sensory block in the chloroprocaine group (p = 0.08). Time to regression differed greatly between the groups with a 6 times faster regression in the chloroprocaine group (Figs. 2 and 3 for regression of motor block and sensory block, respectively, p < 0.001).

**Table 4:** Time to onset and regression of sensory block and motor block (min).

Event	Time to event (min) Median (95% CI)		Log-rank test p-value
	Chloroprocaine 2%—N = 98	Ropivacaine 0.75%—N = 99	
**Sensory block**			
Onset	10.0 (10.0, 15.0)	15.0 (10.0, 15.0)	0.0822
Regression	69.5 (65.0, 75.0)	444.0 (413.0, 475.0)	< 0.0001
**Motor block**			
Onset	10.0 (5.0, 10.0)	10.0 (5.0, 10,0)	0.7911
Regression	65.0 (63.0, 69,0)	405.0 (384.0, 460.0)	< 0.0001

**Figure 4:**
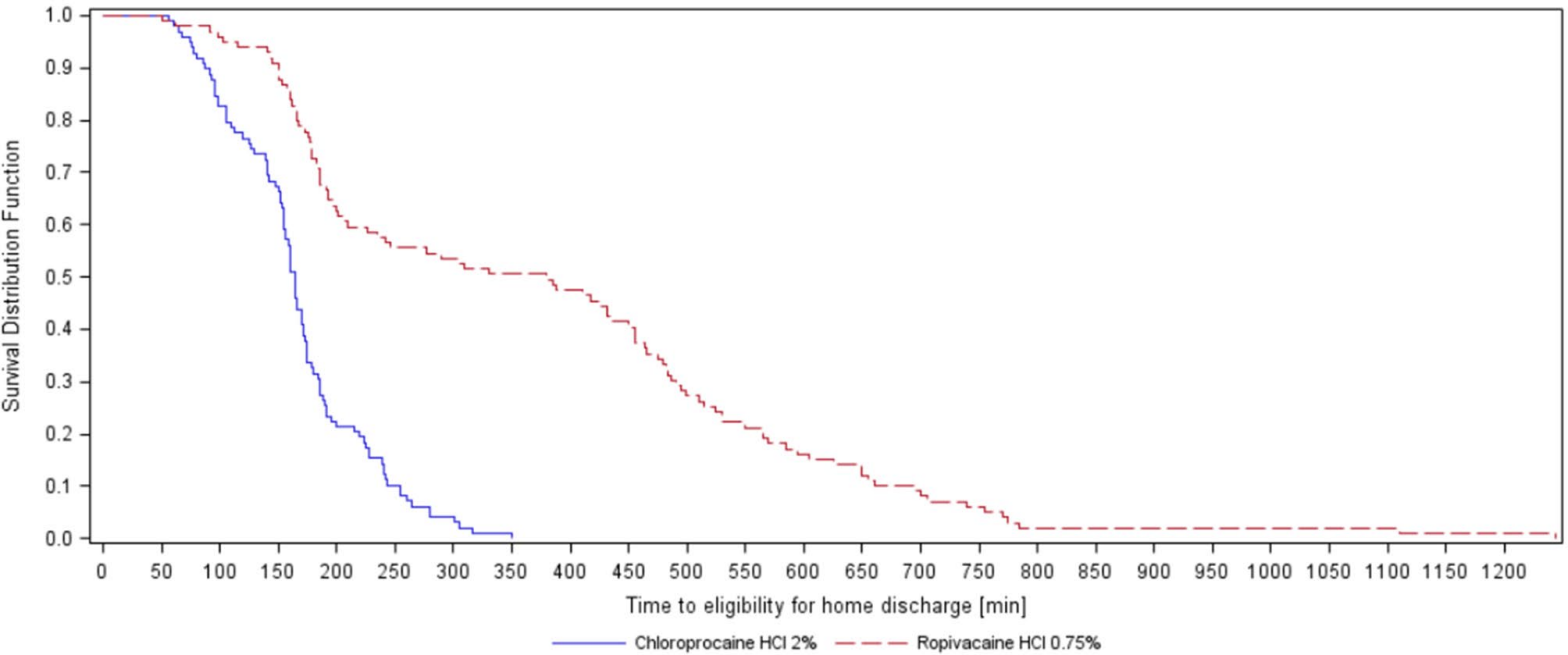
Time to eligibility of home discharge (min).

Time to fulfilment of home discharge criteria was also significantly different, with an earlier discharge for the chloroprocaine group (Fig. 4, p < 0.001): 164.0 (155.0, 171.0) min in the chloroprocaine group vs. 380.0 (209.0, 450.0) min in the ropivacaine group (median, 95% CI). Time to administration of rescue analgesia was not different between the groups (p = 0.6) as was time to first postoperative analgesia (p = 0.11) with 46.7% in the chloroprocaine group and 44.2% in the ropivacaine group requiring postoperative pain medication.

Frequency of neurological symptoms was higher in the ropivacaine vs. the chloroprocaine group for numbness (27 vs. 2 patients), tingling (25 vs. 8 patients), hypoaesthesia (14 vs. 5 patients) and pins/needles sensation (5 vs. 1 patient). Frequency was higher in the chloroprocaine group for aching (10 vs. 6 patients), pricking (8 vs. 5 patients) and burning (8 vs. 1 patient) sensations postoperatively. No clinically relevant differences for ECG or vital sign measurements were observed between the groups.

## Discussion

In the present study chloroprocaine was shown to be non-inferior to ropivacaine in producing a successful nerve block in patients undergoing short duration distal upper limb surgeries with ultrasound guided axillary injection. Onset of sensory or motor block was not significantly different between the groups, however with a tendency for faster onset of sensory block in the chloroprocaine group. Safety was comparable between the two groups and any neurological symptoms exhibited by the patients during the study were not suspicious of neurotoxicity. As was expected by the pharmacological properties of chloroprocaine and ropivacaine regression of sensory and motor blocks was faster with short-acting chloroprocaine compared to ropivacaine, thus allowing a quicker patients' recovery and more rapid fulfilment of the participating hospitals’ discharge criteria by almost 4 h.

Interestingly the primary outcome “*success rate*” of both groups was around 90% and thus slightly lower than the 95% rate that was initially calculated for the sample size analysis, yet still within the range of published success rates^8,14–17^. The difference between the study’s and the published success rates may be attributed to several factors, namely technique, human factor and definition of success—in particular the latter is not universally defined. In the present study, ultrasound was used as per gold standard in all participating hospitals. Each one of the 4 nerves was visualized, identified and blocked separately—a technique, that has proven superior in comparison to single injection techniques, also according to a recent Cochrane analysis^18^. In this analysis, which included 22 trials, it was shown that higher success rates could be achieved with multiple injection techniques in comparison to single injection techniques. However, with regard to safety, where one could hypothetically expect a higher rate of nerve damage with injections close to each nerve separately, evidence remained inconclusive. Success and complete blocks were defined very accurately in the present study with complete sensory block in all four nerve areas as timepoint for “readiness for surgery”^19^—other studies differ as far as definitions of successful block or failure are concerned. In the present study, success was defined as “anaesthesia adequate for the surgery” (*complete* sensory block), *without any supplementation* in the first 45 min (even if surgery lasted for > 45 min). Supplementation was defined as *any* i.v. pain medication or general anaesthesia or pre- or intra-operative systemic analgesia or additional local anaesthetic infiltration. This is a stricter definition in comparison to many other studies, where additional sedation may be allowed^20^, or where a block sufficient for the surgical intervention was defined as successful^21,22^, or where success was not clearly defined at all. In the present study, a sensory block even with one nerve area “not needed” for the surgical intervention was already considered a failed block. Finally in our study only experienced anaesthesiologists performed the procedure, yet they invariably differed with regard to their individual axillary block daily practice^23,24^. Higher or even 100% block success rates may of course be possible, but likely with only a more limited number of anaesthesiologists performing the respective regional anaesthesia^13,16,25^.

With regard to *onset and regression of the nerve blocks*, our results are complementing previous publications describing the unique properties of ropivacaine and chloroprocaine in the setting of regional anaesthesia. Most of these publications date back to pre-ultrasound days. Curiously in a short note by RL Lennon in 1985^26^, chloroprocaine was even suggested as a test substance to assess the right location of an axillary plexus needle placement due to its fast action, with a reported onset of effect of 2–4 min, and a reported absence of toxicity. In the present study, time to onset of motor and sensory block was not significantly different between the groups, however with a distinct tendency for a speedier onset of sensory block for chloroprocaine (p = 0.08). Onset time of sensory and motor block was within the range of previously reported values^27–29^. As expected, difference in regression times and consequently home discharge times were highly significant. The short acting properties of chloroprocaine have also previously inspired trials looking at its cost saving effect, aiming at evaluating the cost-effectiveness of shorter durations of hospital stays due to short acting regional anaesthesia^30,31^, even in comparison to other drugs like lidocaine, also well known for its short action^32^.

Looking at published evidence on chloroprocaine, typically three issues are of major interest: reports of neurotoxicity, rebound pain, and allergy, occurring allegedly more often in ester type regional anaesthetics.

With regard to reports of neurotoxicity of chloroprocaine^33,34^, the present study could not find an increased incidence in the chloroprocaine group, as to be expected from the preservative free formulation. From all treatment *emergent* adverse events, 12 were deemed to be *treatment-related*. These events were all found in the ropivacaine group, consisting mostly of hypoesthesia, as is to be expected after Ropivacaine nerve blocks which may persist up to a mean time of 12 h^35,36^.

With short acting regional anaesthetics in day care surgery, there is the obvious concern of faster pain rebound, when the anaesthetic stops to work earlier than longer acting medications and possible resulting decrease in patient satisfaction^37^. However, there are also reports of dissatisfaction arising typically from prolonged motor blocks, which are associated with an unpleasant sensation. Use of nerve catheters for prolonged nerve blocks is consequently decreasing in many institutions^38,39^. In the present study we did not find any indication of rebound pain, neither was there a difference between the groups for the times until administration of first analgesic, nor was home discharge prevented by postoperative pain. In particular, the earlier regression of sensory and motor block occurring with chloroprocaine, allows for early home discharge without any residual anaesthesia unlike what happens when long acting local anaesthetics are used. This can represent a valuable benefit for the patients’ safety to avoid domestic incidents, e.g. additional injuries of the anaesthetized extremity^40,41^, once the patients are at home and are no longer under the direct supervision of a professional health care provider.

Finally aminoester class local anaesthetics have been reported to have higher allergy rates than aminoamid class local anaesthetics—an idea that is propagated in textbooks, albeit with very moderate evidence^42^. In a recent study in 177 patients only erythema after subcutaneous infection was visible as to be expected by the vasodilating properties of ester linked regional anaesthetics but no evidence of any type-1 allergenicity was found^43^.

The study has some limitations. The comparison with a particularly long acting drug may seem uncommon. However, ropivacaine was chosen not for its similar pharmacodynamic properties but for its use as most commonly used substance in the setting of a pivotal study. Secondly the study’s primary outcome was “only” non-inferiority with regard to successful block and not e.g. superiority for home discharge. Yet, since this was a pivotal study, use of successful block as non-inferior primary outcome was invariably chosen, according to the requirements of European Medicines Agency as described at the beginning of the method section. Also it would have been interesting to assess patient satisfaction more in detail, since reports differ regarding the impact of long and short motor block on patient satisfaction^37^. Finally the study only included only minor surgeries, in which for any regimen low postoperative pain scores may be expected and consequently low post-operative analgesia requirements, and surgeries were not standardized to one type of surgery but were heterogenous**.**

In summary, the present study shows that for short-duration surgical procedures (60 min) Chloroprocaine HCl 2% may be used with success rates non-inferior to Ropivacaine HCl 0.75%; Chloroprocaine HCl 2% features a favorable safety profile and a rapid remission of sensory and motor block.

## Supplementary Information

Supplementary Information.

## Data Availability

The datasets used and/or analyzed during the current study are available from the corresponding author on reasonable request.
