# Supplementary material for: A randomised, non-inferiority study of chloroprocaine 2% and ropivacaine 0.75% in ultrasound-guided axillary block
Source: Sci Rep. 2021 May 11;11:10035. doi: 10.1038/s41598-021-89483-y (PMC8113228; doi:10.1038/s41598-021-89483-y)
Supplement: Supplementary file 1 — Supplementary Information. [file 41598_2021_89483_MOESM1_ESM.docx]

| **Electronic supplement 1 – study schedule** |  |
| --- | --- |

| **ACTIVITIES** | **Visit 1 -  Screening** | **Visit 2 -  Treatment** | **Post-operative  recovery,  final visit** | **Telephonic  Follow-up** |
| --- | --- | --- | --- | --- |
| **Visit** | **Days -14/1** | **Day 1** | **Day 1/2** | **Day 7±1** |
| **Informed consent** | x |  |  |  |
| **Demography** | x |  |  |  |
| **Medical/surgical history** | x |  |  |  |
| **Physical abnormalities** | x |  |  |  |
| **Previous and concomitant medications** | x | x | x | x |
| **Height** | x |  |  |  |
| **Body weight** | x |  |  |  |
| **Vital signs**  **(blood pressure, heart rate)^1^** | x | x | x |  |
| **SpO2^2^** |  | x | x |  |
| **ECG^1^** |  | x | x |  |
| **Pregnancy test (urine)** | x |  |  |  |
| **Eligibility evaluation** | x | x |  |  |
| **Enrolment and randomisation^3^** |  | x |  |  |
| **Oral midazolam premedication (if necessary)^4^** |  | x |  |  |
| **Anaesthesia (investigational product administration)^5^** |  | x |  |  |
| **Sensory block assessment^6^** |  | x |  |  |
| **Motor block assessment^6^** |  | x |  |  |
| **Surgery (< 60 min)** |  | x |  |  |
| **Aldrete’s scoring scale^7^** |  |  | x |  |
| **Home discharge^7^** |  |  | x |  |
| **Meals^9^** |  |  | x |  |
| **Adverse events monitoring^8^** | x | x | x | x |
| **Neurological symptoms questionnaire** |  |  | x | x |

1. *Vital signs at screening; vital signs and ECG (if foreseen by the standard hospital procedures) at baseline, during the block until the end of the anaesthesia and during post-operative recovery (final visit)*
2. *SpO2 before the block (baseline), during the block until the end of the anaesthesia and during post-operative recovery (final visit)*
3. *On day 1, before any study procedures subjects were randomised to either Chloroprocaine HCl 2% or Ropivacaine HCl 0.75% treatment group*
4. *Oral premedication with midazolam was allowed, if necessary*
5. *Patients received anaesthetic block with either Chloroprocaine or Ropivacaine, before surgery, according to the randomisation list and parallel-group design*
6. *Sensory and motor blocks were assessed after block placement, every 5 min until the patient was ready for surgery, as soon as possible after surgery, then every 15 min for the first hour after surgery, every 30 min for the next 2 h and then every 1 h until regression of the blocks. The reference time point for calculating the scheduled time interval of the post-surgery assessments is the time of surgery end*
7. *Patients were discharged on Day 1 or on a following day after the criteria for discharge were met and according to the hospital’s standard procedures. In case of discontinuation, subjects underwent an early termination visit (ETV)*
8. *AEs monitored from the screening visit, immediately after informed consent signature, up to the telephonic follow-up. Particular attention was given to systemic and local toxicity symptoms, neurological symptoms (e.g. paraesthesia, motor function problems and pain at the injection site) and allergic reactions.*
9. *Meals were served according to the hospital’s standard procedures*
